# Genetic Structure of the Rice Blast Pathogen (*Magnaporthe oryzae*) over a Decade in North Central California Rice Fields

**DOI:** 10.1007/s00248-017-1029-4

**Published:** 2017-07-28

**Authors:** Deborah Pagliaccia, Ryan Z. Urak, Frank Wong, LeAnn I. Douhan, Christopher A. Greer, Georgios Vidalakis, Greg W. Douhan

**Affiliations:** 1 Department of Plant Pathology and Microbiology, University of California, Riverside, CA 92521 USA; 2 Present Address: Department of Botany and Plant Sciences, University of California, Riverside, CA USA; 3 Present Address: Bayer’s Environmental Health Division, Bayer, Durham, NC USA; 4 Present Address: Val Verde Unified School District, Perris, CA USA; 5 Cooperative Extension, University of California, Sutter-Yuba, Yuba City, CA 95991 USA; 6 Present Address: Cooperative Extension Tulare County, Tulare, CA USA

**Keywords:** Rice blast disease, Population structure, *Pi-z* gene, AFLP, California, *Pyricularia oryzae*

## Abstract

Rice blast, caused by the ascomycete *Magnaporthe oryzae*, is one of the most destructive rice diseases worldwide. Even though the disease has been present in California since 1996, there is no data for the pathogen population biology in the state. Using amplified fragment length polymorphisms and mating-type markers, the *M. oryzae* population diversity was investigated using isolates collected when the disease was first established in California and isolates collected a decade later. While in the 1990 samples, a single multilocus genotype (MLG) was identified (MLG1), over a decade later, we found 14 additional MLGs in the 2000 isolates. Some of these MLGs were found to infect the only rice blast-resistant cultivar (M-208) available for commercial production in California. The same samples also had a significant decrease of MLG1. MLG1 was found infecting the resistant rice cultivar M-208 on one occasion whereas MLG7 was the most common genotype infecting the M-208. MLG7 was identified in the 2000 samples, and it was not present in the *M. oryzae* population a decade earlier. Our results demonstrate a significant increase in genotypic diversity over time with no evidence of sexual reproduction and suggest a recent introduction of new virulent race(s) of the pathogen. In addition, our data could provide information regarding the durability of the *Pi-z* resistance gene of the M-208. This information will be critical to plant breeders in developing strategies for deployment of other rice blast resistance genes/cultivars in the future.

## Introduction

Rice (*Oryza sativa* L.) is an important agricultural commodity that supplies approximately 30% of the nutritional intake of the world’s population [1]. One of the most important diseases of rice globally is rice blast, caused by the ascomycete fungus *Magnaporthe oryzae* B.C. Couch (syn. *Pyricularia oryzae* Cavara), which causes up to 20% yield loss in many production zones annually and up to 80–100% yield loss when significant epidemics occur [2, 3]. *M. oryzae* can infect most parts of the plant, but infections of the panicle neck node or the panicle are the most damaging phases of the disease [2] with reports of the disease occurring in more than 85 countries [4]. When *M. oryzae* infects rice and produces symptoms of neck rot or panicle blast, normal panicle and seed development is inhibited. Additional closely related species, such as *Magnaporthe grisea* (anamorph *Pyricularia grisea*), also cause similar diseases in other graminaceous species including crop plants such as wheat and millet as well as cause significant damage to various turf grass species [2, 5–7].

*M. oryzae* was first identified in the rice-producing regions of north central California in 1996 [8]. This was unexpected due to the pathogen’s common association with high humidity conditions [9], which is unlike the temperate Sacramento Valley. Seeds, crop residue, and secondary hosts are all possible vehicles [4, 10–12] for the introduction of *M. oryzae* into California and could have been the primary sources of inoculum for the disease. Since it was first reported, rice blast has become an endemic disease of rice on the west side of the Sacramento Valley and can cause significant yield loss in some fields when environmental conditions, agronomic practices, and rice variety selections favor disease development (Greer, personal observation). Farming practices that minimize the risk of disease development in conjunction with the usage of fungicides and deployment of resistant cultivars are currently the best courses of action to manage the disease. The disease is mainly controlled by azoxystrobin fungicide applications and to a much lesser extent by the cultivation of the rice blast-resistant variety M-208 with the single resistance gene *Pi-z* [13]. M-208 was developed as a Calrose medium grain rice variety resistant to the IG-1 race of the rice blast pathogen that was originally introduced into California [13]. M-208 was first grown commercially in 2006, and resistance to rice blast was stable until very limited neck blast lesions were observed in some M-208 fields during the 2010 and 2011 growing seasons (Greer, personal observation).

*M. oryzae* is a heterothallic fungus with a single mating-type gene that produces two alleles, MAT1-1 and MAT1-2. The pathogen requires both mating types in order for sexual reproduction to occur [14]. With respect to rice production, limited studies have provided evidence that sexual reproduction may be occurring under field conditions in some parts of the world. Kumar et al. [15] failed to reject random mating based on multilocus disequilibrium tests in populations of *M. oryzae* collected in the Himalayan region of India. Likewise, Saleh et al. [16] detected linkage disequilibrium in a population of *M. oryzae* from Yunnan providence, China, and also found that isolates from that population could produce viable progeny in vitro when crosses were made. Moreover, Tharreau et al. [17] analyzed more than 1700 isolates from 40 countries using 13 microsatellite markers and found three main groups based on principal component analysis; one group contained only MAT1-1 isolates, another group contained only MAT1-2 isolates, and a third group contained both mating types. The group that contained both mating types was also the most genetically diverse and contained many isolates from the countries of the Himalayan foothills, consistent with the work of Kumar et al. [15] and Saleh et al. [16], suggesting that this is the center of origin of *M. oryzae* and that sexual reproduction may be occurring in this region in contrast to other production areas where asexual reproduction dominates.

The first objective of this research was to analyze the genetic structure of *M. oryzae* in California from isolates collected from the initial identification of the disease in the mid-1990s to more recently sampled isolates (2007 and 2010) using amplified fragment length polymorphisms (AFLP) and mating-type molecular markers. This would shed light on whether or not any changes in the genetic structure of the fungal population have occurred over a decade which may be linked to fungicide usage, resistant rice cultivar deployment, new pathogen introductions, or reproductive mode (sexual and/or asexual) occurring under field conditions. Given the recent findings that the previously released resistant rice cultivar M-208 is now experiencing blast symptoms in California, the second objective of this study was to determine if these new *M. oryzae* isolates (2010) represent distinct genotypes which could suggest a second, more recent introduction of new virulent race(s) of the pathogen. Alternatively, if these new isolates were similar or have the same genotypes to the older isolates (1990s), this could provide information regarding the durability of the *Pi-z* resistant gene which is currently the only commercially available source of resistance to rice blast in California. This information will be critical to plant breeders in developing strategies for deployment of other rice blast resistance genes/cultivars in the future.

## Materials and Methods

### Sampling

**Fig. 1:**
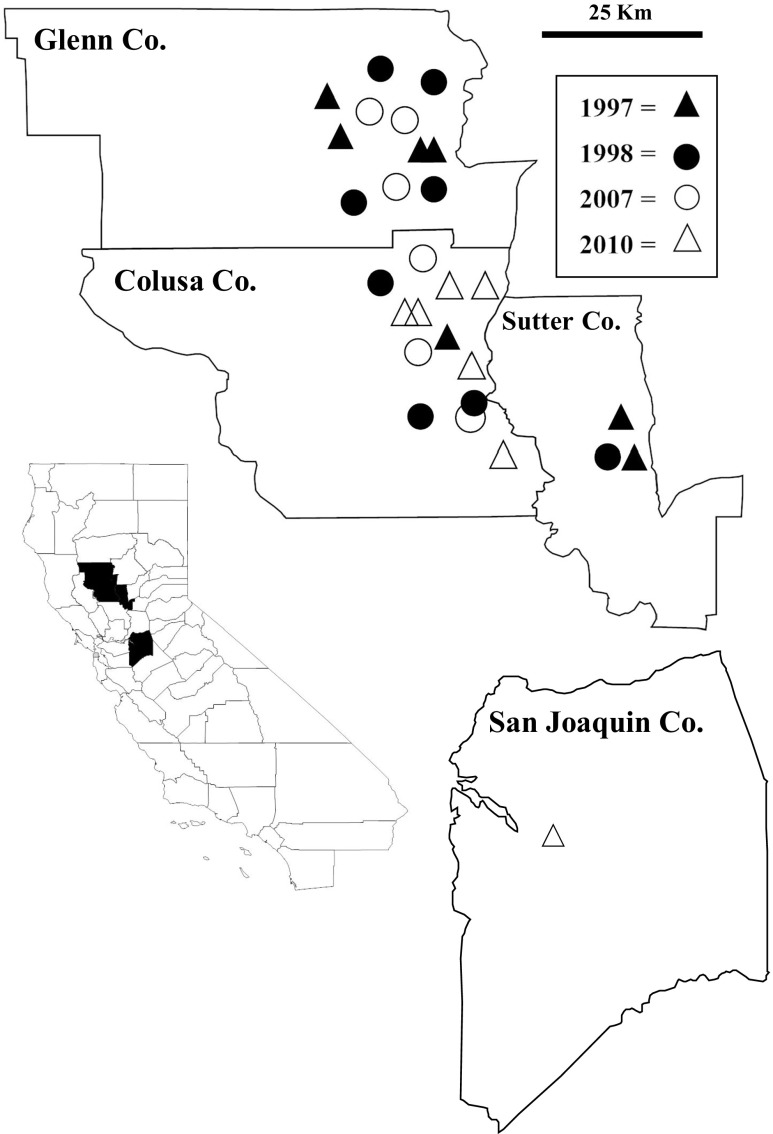
Distribution of sampled *Magnaporthe oryzae* isolates collected from 28 *Oryza sativa* fields used in this study. The map of California shows the four counties sampled which are expanded to show the spatial distribution among the sampled locations. Note that the San Joaquin county is not in proportion to the *scale bar* in the north by south direction and the distance between the southernmost sampled locations in the Sutter county to the single sampled location in the San Joaquin county is approximately 100 km. The cultivars sampled in 1997, 1998, and 2007 were all susceptible and were likely M-201 and M-202 (exact cultivar identifications could not be made). In 2010, all of the fields sampled were from the resistant cultivar M-208 except for the single field in the San Joaquin county which was sampled from the susceptible cultivar M-104

*M. oryzae* isolates from 1997 and 1998 were obtained from long-term storage from Dr. Tom Gordon, Department of Plant Pathology, University of California, Davis; these were originally isolated and described by Greer and Webster [8] and were collected from the Glenn, Colusa, and Sutter counties of California (Fig. 1). In 2007, six rice fields were sampled from the Glenn and Colusa counties in locations similar to the 1997 and 1998 collections (Fig. 1). Plants were sampled by randomly collecting diseased panicle tissues exhibiting typical rice blast lesions along a single transect (~30 m) approximately 5 m from the edge of the rice fields. The tissues were placed in paper bags, brought back to the laboratory, and air-dried in the fume hood for several days.

In the 2010–2011 growing seasons, rice blast was observed for the first time in rice fields planted with the resistant rice cultivar M-208. Fields planted with the M-208 variety were randomly sampled throughout the rice-growing region of California in search of infected plant material. Six fields (Fig. 1) were found to have rice blast-symptomatic plants, and diseased panicles were collected and processed as above. In the same growing seasons, rice blast was also found for the first time in one rice field growing the susceptible cultivar M-104 west of Stockton in the San Joaquin county which is approximately 100–150 km south of the main northern production area where rice blast occurs regularly and diseased panicles were collected and processed as above (Fig. 1). One isolate of *P. grisea* from *Pennisetum clandestinum* Hochst. ex Chiov. (kikuyu grass) was also used in the analysis for comparative purposes since host specificity has been documented among various grass species [18].

### Fungal Isolation and DNA Extraction

To isolate *M. oryzae*, the infected tissue was surface-sterilized using 5% bleach and briefly rinsed in sterile water, and the lesions were cut in half. The tissues were placed in petroleum jelly that was positioned on the lids of acid potato dextrose agar (APDA) plates (Fisher Scientific, USA) so that they would be elevated. After 48 h, the lids were tapped to release the spores and the plates were allowed 4–7 days for growth at room temperature. Five germinated single spores were randomly selected and removed from the APDA plates and were then transferred to clean APDA plates, and a single isolate was recultured to produce a single isolate per sample. PDA was also used to regrow the 1997 and 1998 collections of *M. oryzae* isolates from long-term storage.

The isolates were allowed to grow at room temperature for approximately 1 week before mycelium, and spores were scraped off each plate using a sterile spatula and placed into a cetyltrimethylammonium bromide (CTAB) extraction buffer (2% CTAB, 100 mM Tris, pH 8.4, 10 mM EDTA, and 0.7 M NaCl) [19]. Total DNA was extracted using chloroform, precipitated using 70% ethanol and 3 M sodium acetate [20], and resuspended in 100 μl of TE buffer (10 ml of Tris, pH 8.0, 1 Mm EDTA). To evaluate the relative quantity and quality of the genomic DNA, 5.0 μl of each extraction was stained using SYBR® Green I (Thermo-Fisher Scientific, USA) following the manufacturer’s instructions, separated in a 0.8% agarose gel, and visualized under UV light.

### Amplified Fragment Length Polymorphisms

The method of Vos et al. [21] was used to develop the initial restriction-ligation products used for AFLP amplification, but fluorescently labeled primers were used to resolve the markers via a capillary sequencer as described below. A total of eight randomly chosen primer combinations with the addition of two or three selective nucleotides at the 3′ end were used to screen a subset of eight isolates, and two primer combinations were chosen for the final analysis which resolved the fragment patterns well under the experimental conditions used. The two chosen primer combinations were FAM ECO RI + GC/MSE I + GA and HEX ECO RI + TA/MSE I + CG. Each 20-μl reaction contained 1× PCR buffer (Invitrogen, Carlsbad, CA), 2.5 mM MgCl_2_, 2.5 mM each dNTP (Invitrogen), 0.375 μM of each primer, 0.5 U of Taq polymerase (Invitrogen), and 10 μl of template of the restriction ligation dilution. Nine *M. oryzae* isolates were used as controls by conducting independent DNA extractions, restriction-ligation reactions, and PCR amplifications. PCR thermocycling conditions consisted of an initial hold at 72 and 94 °C for 1 and 4 min, respectively, followed by 44 cycles with an annealing temperature of 65 °C for 30 s and an extension temperature of 72 °C for 1 min with the annealing temperature reduced by 1 °C for the first 9 cycles. All amplifications were performed in a MyCycler (Bio-Rad Laboratories Inc., USA).

The labeled FAM and HEX fragments were detected using an ABI 3100 16-capillary instrument at the CORE Instrumentation Facility at UC Riverside. The Genographer 2.0 fragment analysis software was used to score the data by setting the intensity settings between 1 and 5 and manually scoring the presence or absence of clearly visible polymorphic bands above 100 bp by checking chromatograms as well as an electronic image of the gel. Putative alleles at each polymorphic AFLP locus were scored using a binary code (1 and 0) corresponding to positive and null alleles, respectively. For the selection of AFLP markers, only polymorphic loci that were reproducible and easily identifiable were chosen.

Cluster analysis on genetic distances using the mean character difference option and unweighted pair-group method with arithmetic averaging (UPGMA) were conducted using PAUP* (version 4.0 beta 10). Neighbor-joining (NJ) and maximum parsimony (MP) analyses using PAUP were also conducted which produced similar results, so only the UPGMA analyses are described. The analysis was conducted twice; once using all isolates so that putative clones could be identified by location, and then, the data set was reduced to a single representative of each genotype identified. Confidence in tree topology was examined using bootstrap with 10,000 replicates.

### Mating-Type Designation

The mating type of each isolate was identified using the mating-type-specific primers B16 and B182 for MAT1-1 and A1 and A5 for MAT1-2 [22, 23]. The expected PCR products were 371 bp for MAT1-1 and 666 bp for MAT1-2. Two microliters of genomic DNA diluted (1:100) in sterile water was used as template in a 20 μl PCR mixture. One multiplex PCR amplification was performed with the four mating-type-specific primers. Each 20-μl PCR reaction contained 1× PCR buffer (Invitrogen), 0.2 mM dNTP (Invitrogen), 3.75 μM of each primer, and 0.5 unit of Taq polymerase. All amplifications were performed in a MyCycler (Bio-Rad Laboratories Inc). PCR thermocycling conditions consisted of an initial hold at 95 °C for 3 min, followed by 35 cycles of 95 °C (30 s), 55 °C (30 s), and 72 °C (1 min). The PCR products were then stained and visualized as previously described using 1.5% agarose gels.

## Results

**Fig. 2:**
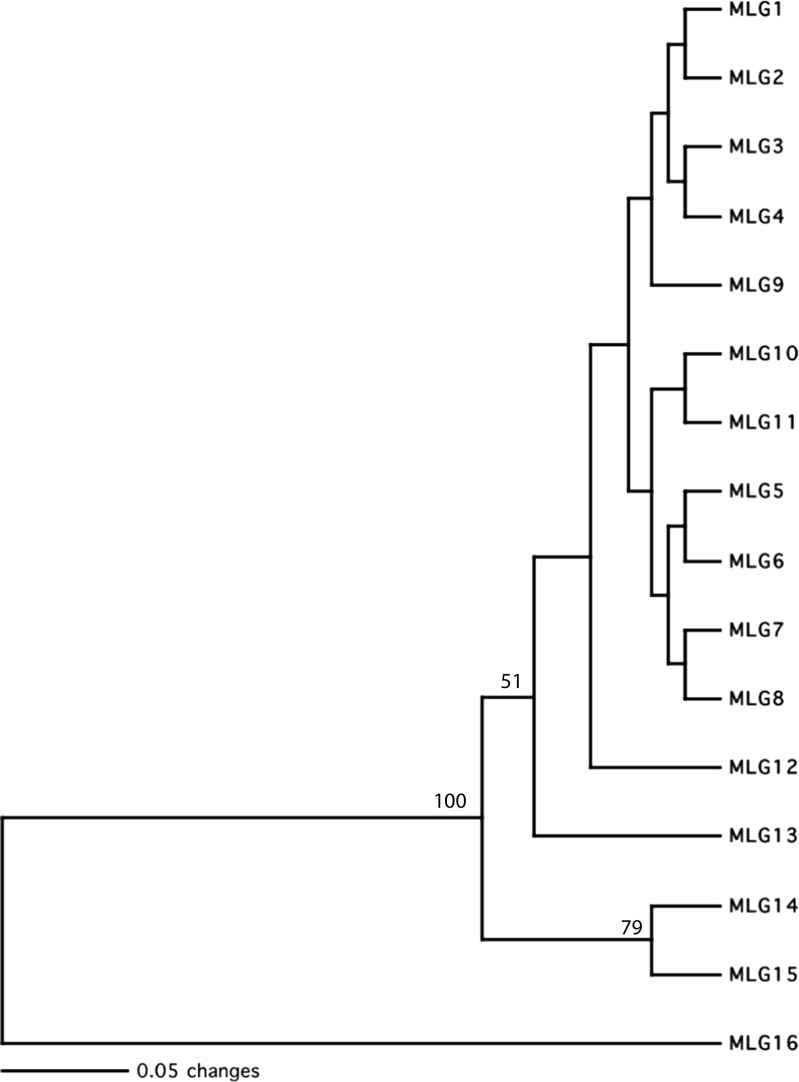
Dendrogram of multilocus genotypes (MLGs) of *M. oryzae* (MLGs 1–15) and *Pyricularia grisea* (MLG16) based on UPGMA cluster analysis using PAUP* with genetic distances calculated from 37 AFLP loci. Bootstrap values are based on 10,000 replicates with values over 50% shown (see Table 1 for information regarding the distribution of the MLG from the sampled *Oryza sativa* fields)

A total of 193 *M. oryzae* isolates collected from 28 rice fields were analyzed in this study; 29 collected from 7 fields in 1997, 33 collected from 8 fields in 1998, 97 collected from 6 fields in 2007, and 34 collected from 7 fields in 2010 (Table 1). The cultivars sampled in 1997, 1998, and 2007 were all susceptible M-201 and M-202 cultivars. Although exact cultivar identifications could not be made from the specific fields sampled, both cultivars have no resistance to rice blast (M-202 was created to replace cultivar M9 in cooler areas where M-202 threshed harder than M-201). All of the fields sampled in 2010 were from the resistant cultivar M-208 except for the single field in the San Joaquin county which was sampled from the susceptible cultivar M-104. A total of 37 polymorphic AFLP loci were scored, and the controls produced identical results (data not shown). Within the rice-specific isolates, 14 of the scored loci were polymorphic with the remaining markers being host-specific to the kikuyu grass isolate. There were a total of 15 multilocus genotypes (MLGs) identified from the rice isolates and a 16th genotype that was isolated from kikuyu grass (Fig. 2). As previously determined [18], the kikuyu grass isolate was significantly divergent from the rice isolates, which all clustered together with 100% bootstrap support. Within the rice isolates, MLGs 14 and 15 clustered together with 79% whereas the rest of the MLGs clustered together with only 51% bootstrap support.

**Table 1:** Summary of multilocus genotypes (MLGs) of *Magnaporthe oryzae* isolated from 28 *Oryza sativa* fields used in this study

Year isolated	Field code^a^	MLG															Total No. of isolates sampled per year	Total No. of genotypes sampled per field
		1	2	3	4	5	6	7	8	9	10	11	12	13	14	15		
1997	97-1	5																1
	97-2	1																1
	97-6	7																1
	97-21	4																1
	97-63	4																1
	97-64	3																1
	97-69	5															29	1
1998	98-1	7																1
	98-4	2																1
	98-10	5																1
	98-12	2																1
	98-14	3																1
	98-15	5																1
	98-17	4																1
	98-19	5															33	1
2007	07-1	13	1					1										3
	07-2	16						2										2
	07-3	2						1	1									3
	07-4	13	1	1				7										4
	07-5	11			1	1		3										4
	07-6	2	18				1			1							97	4
2010	10-1							4										1
	10-2	1						3										1
	10-3							1					1		1	1		4
	10-4							2						1				2
	10-5							10										1
	10-6							1			1	1						3
	10-7	2						4									34	2
Total No. of individual MLGs sampled		122	20	1	1	1	1	39	1	1	1	1	1	1	1	1	193	

^a^For the 1997, 1998, and 2007 locations, *M. oryzae* was isolated from susceptible cultivars which were most likely M-201 and M-202. Although exact cultivar identifications could not be made from the specific fields sampled, both cultivars have no resistance to rice blast (M-202 was created to replace cultivar M9 in cooler areas where M-202 threshed harder than M-201). *M. oryzae* was isolated in 2010 from the resistant cultivar M-208 for fields 10-1 to 10-6 and the susceptible cultivar M-104 for field 10-7

MLG1 was the only genotype found within the 1997 and 1998 samples and was also dominant in the 2007 samples. However, in the 2010 isolates obtained primarily from the resistant rice cultivar M-208, MLG1 was not dominant and was only found three times whereas MLG7 dominated the samples from 2010 and accounted for 25 of the 34 collected isolates (Table 1). This same genotype was also present in the 2007 collection but only accounted for 14 of the 97 isolates that were collected in 2007. The only other dominant genotype that was found was MLG2, which was found (*n* = 20) only in the 2007 collection. The rest of the identified genotypes was found only once in multiple locations from the 2007 and 2010 collections of isolates (Table 1). There was an obvious increase of *M. oryzae* genotypic diversity from 1997/1998 to 2010 since only MLG1 was found in 1997/1998, whereas 14 additional genotypes were discovered in the 2007 and 2010 isolate collections. However, MLG1 was also isolated from one field growing the resistant rice cultivar M-208. The mating-type PCR assay identified a single mating type (MAT1-2) in the entire collection of *M. oryzae* isolates.

## Discussion

This is the first study to investigate diversity of the *M. oryzae* population in California using a large sample size of isolates and including isolates collected over a decade of time. All isolates belonged to a single mating-type (MAT1-2). Only a single genotype (MLG1) was found in 1997 and 1998, which was only 2 years after rice blast was first identified in California. Over a decade later, 14 additional genotypes of *M. oryzae* were identified. However, MLG1 was still the dominant genotype found on susceptible rice cultivars while two additional genotypes were also common (MLG2 and MLG7). In contrast to 2010 when isolates were collected only from the resistant rice cultivar M-208, MLG1 was found only once in one field whereas MLG7 was still common, demonstrating that both MLGs were pathogenic to M-208 in addition to six more MLGs. Even though our data did not provide any insight on the origin of the new genotypes or differentiated if they arose by mutation within the original genotype, our results clearly demonstrate that *M. oryzae* is not sexually reproducing but there has been a significant increase in genotypic diversity over time in California. Further work, based on sequencing data from a large population and additional genetic analysis paired with pathogenicity tests, using an extended differential cultivar set may answer the question of the origin of *M. oryzae* genotypes in California.

In this study, only a single mating type of *M. oryzae* was found in California rice fields, which is consistent with many other studies evaluating mating-type distribution within rice populations of *M. oryzae*. For example, Consolo et al. [22] analyzed 125 isolates from Argentina and Park et al. [23] analyzed 254 isolates from Korea, and all were found to belong to the MAT1-1 mating type. A predominance of a single mating type has also been identified in other geographic regions such as in Vietnam, Thailand, the Philippines, Europe, and in some regions of India [24–26]. However, in the USA, both mating types have been identified in Arkansas, Mississippi, Texas, and Missouri but there have been no studies in the USA on the population structure of *M. oryzae* associated with rice that has demonstrated the potential that sexual reproduction is occurring in the USA [27]. The only rice-producing areas where sexual reproduction may be occurring are within the center of origin of the pathogen, and most other studies globally on rice isolates have consistently described a clonal population structure.

Although increased genotypic diversity was observed in this study from isolates collected over a decade apart, only one to four genotypes and one mating type of *M. oryzae* were identified from each field sampled. This suggests that *M. oryzae* associated with rice is not sexually reproducing in California, which can be important in the population dynamics of this pathogen. Sexual reproduction can reshuffle genetic material via recombination, thereby bringing together new alleles, which may influence pathogenicity to various rice cultivars and could influence the efficacy of fungicides. However, genetic exchange within *M. oryzae* could potentially occur via heterokaryosis and/or the parasexual cycle as demonstrated by Noguchi et al. [28]. Additionally, auxotrophic mutants of *M. oryzae* have been used to demonstrate segregation of morphological and nutritional markers [29, 30] and Zeigler et al. [31] used molecular markers to demonstrate the detection of parasexual DNA exchanges in unselected wild-type strains.

Despite evidence of relatively even mating types, gametic equilibrium, and successful in vitro crosses from isolates from within the putative origin of *M. oryzae*, perithecia have yet to be discovered under field conditions so the role of ascospores in creating diversity and in dissemination of the pathogen is still elusive despite many years of studying the population biology of this important pathogen [32]. Given the fact that there are lineages associated with isolates belonging to one or the other mating type and areas of mixed mating types, it has been hypothesized that *M. oryzae* has lost its ability to sexually reproduce due to the rapid spread and domestication of rice and potentially founding populations of the pathogen which have followed this domestication [26]. Not only are both mating types needed for sexual reproduction to occur in *M. oryzae*, but there is also a necessity in having strains that are female-fertile in order for perithecia to develop. Seleh et al. [33] demonstrated in the laboratory that female fertility can be lost in vitro in *M. oryzae* rice strains when they are grown under asexual conditions. However, in other species or isolates associated with other grass hosts, there has been evidence that sexual reproduction may be occurring [18, 34].

To complicate matters, the fungi that cause blast or gray leaf spot diseases of grasses were first described as the anamorphic species *P*. *grisea*, but once the sexual state was identified, the name was changed to *M. grisea* [35]. However, the taxonomic status of these fungi associated with these diseases of rice and other grasses has been the source of considerable debate (see Tosa and Chuma [35] for a recent review).

Host selection has been one of the major driving forces in the speciation process of these fungi, and Couch and Kohn [36] were the first to use molecular data to support earlier studies that suggested that isolates associated with rice blast should be referred to as *M. oryzae*. In a later study, Couch et al. [37] determined the genetic relationship between rice isolates and isolates of other rice-associated grass hosts and suggested a single fungal invasion of rice followed by shifts to other grass hosts that grew in association with cultivated rice. Douhan et al. [18] investigated the diversity of *P. grisea* isolates from five turf species as well as isolates from rice (*M. oryzae*) and found well-supported clades based on host preference consistent with other studies. However, Klaubauf et al. [38] have recently conducted a multilocus phylogeny on a global collection of *Pyricularia*/*Magnaporthe* isolates and determined that certain host genera (*Eleusine*, *Oryza*, *Setaria*, and *Triticum*) were exclusively infected with isolates belonging to *M. oryzae* while other hosts (*Cenchrus*, *Echinochloa*, *Lolium*, *Pennisetum*, and *Zingiber*) were infected by different species and sometimes more than one species, demonstrating that host preference alone cannot resolve species relationships with pathogens associated with blast or gray leaf spot diseases.

In California, the severity of rice blast has decreased since the initial 1996 introduction because, as previously postulated, *M. oryzae* cannot flourish in the environmental conditions that normally exist in California [8]. California rice production takes place in a climate that is permissive for rice blast but is too arid to allow the onset of significant epidemics in most years. Therefore, management is still needed when conditions are favorable for the disease [8]. Growers in other parts of the world primarily use resistant rice cultivars [2], but fungicides are also used when disease pressure is significant. Azoxystrobin is the standard and consequently the most widely used fungicide for rice blast in California [8]. Azoxystrobin effectively inhibits spore germination and is therefore a protectant prior to infection [39].

The resistant rice cultivar M-208 and a single fungicide have been the primary means to managing rice blast in California during the past decade. Our results, however, demonstrate that under California conditions, reliance on a single source of resistance will not be effective to control rice blast since the “original” genotype (MLG1) can infect rice with the *Pi-z* gene (M-208), the common MLG7 was found in all fields growing M-208 in 2010, and the population of *M. oryzae* is now more diverse compared to its initial introduction. This problem is particularly important for newly released rice cultivars for commercial use in California such as M-209 because its field resistance to rice blast is not known [40]. Therefore, due diligence to breed more resistance into rice cultivars for California is needed and breeders need to be aware of the pathogen diversity when screening germplasm for resistance. Further research is also needed to investigate alternative fungicides and/or management practices to more effectively manage rice blast under California conditions.

## References

[1] Gnanamanickam SS (2009). Rice and its importance to human life. Biological control of rice diseases.

[2] Ou SH (1985) Rice diseases, 2nd edn. Commonwealth Mycological Institute, Kew, Surrey, UK

[3] Prabhu AS, Filippi MC, Silva GB, Lobo VLS, Moraes OP, Wang GL, Valente B (2009). An unprecedented outbreak of rice blast on a newly released cultivar BRS Colosso in Brazil. Advances in genetics, genomics and control of rice blast disease.

[4] Agarwal PC, Mortensen CN, Mathur SB (1989) Seed-borne diseases and seed health testing of rice. Tech. Bull. No. 3, Phytopathological Papers No. 30 CAB International Mycological Institute, Kew, UK

[5] Bain DC, Patel MV, Patel BN (1972). Blast of ryegrass in Mississippi. Plant Dis Rep.

[6] Isaac MM, Owen JH (1957). The grayleaf-spot disease of St. Augustine grass. Plant Dis Rep.

[7] Sundaram NV, Palmer LT, Nagarajan KN, Prescott JM (1972). Disease survey of sorghum and millet in India. Plant Dis Rep.

[8] Greer CA, Webster RK (2001). Occurrence, distribution, epidemiology, cultivar reaction, and management of rice blast disease in California. Plant Dis.

[9] Webster RK, Gunell PS (1992). Compendium of rice diseases.

[10] Lee FN, Zeigler RS, Leong SA (1994). Rice breeding programs, blast epidemics and blast management in the United States. Rice blast disease.

[11] Rao KM (1994). Rice blast disease.

[12] Teng PS, Zeigler RS, Leong SA (1994). The epidemiological basis for blast management. Rice blast disease.

[13] Johnson CW (2008) Rice cultivar M-208 (US patent; application number 20080196117)

[14] Yoder OC, Valent B, Chumley F (1986). Genetic nomenclature and practice for plant pathogenic fungi. Phytopathology.

[15] Kumar J, Nelson RJ, Zeigler RS (1999). Population structure and dynamics of *Magnaporthe grisea* in the Indian Himalayas. Genetics.

[16] Saleh D, Xu P, Shen Y, Li CG, Adreit H, Milazzo J, Ravigne V, Bazin E, Notteghem JL, Fournier E, Tharreau D (2012). Sex at the origin: an Asian population of the rice blast fungus *Magnaporthe oryzae* reproduces sexually. Mol Ecol.

[17] Tharreau D, Fudal I, Andriantsimialona D, Santoso UD, Fournier E, Lebrun MH, Notteghem JL (2009). World population structure and migration of the rice blast fungus, *Magnaporthe oryzae*.

[18] Douhan GW, de la Cerda KA, Huryn KL, Greer CA, Wong FP (2011). Contrasting genetic structure between *Magnaporthe grisea* populations associated with the golf course turfgrasses *Lolium perenne* (perennial ryegrass) and *Pennisetum clandestinum* (Kikuyugrass). Phytopathology.

[19] Gardes M, Bruns TD (1993). ITS primers with enhanced specificity for Basidiomycetes—application to the identification of mycorrhizae and rusts. Mol Ecol.

[20] Lee SB, Taylor JW, Innis MA, Gelfand DH, Sninsky JJ, White TJ (1990). Isolation of DNA from fungal mycelia and single spores. PCR protocols: a guide to methods and applications.

[21] Vos P, Hogers R, Bleeker M, Reijans M, Vandelee T, Hornes M, Frijters A, Pot J, Peleman J, Kuiper M, Zabeau M (1995). AFLP—a new technique for DNA-fingerprinting. Nucleic Acids Res.

[22] Consolo VF, Cordo CA, Salerno GL (2005). Mating-type distribution and fertility status in *Magnaporthe grisea* populations from Argentina. Mycopathologia.

[23] Park SY, Milgroorn MG, Han SS, Kang S, Lee YH (2008). Genetic differentiation of *Magnaporthe oryzae* populations from scouting plots and commercial rice fields in Korea. Phytopathology.

[24] Notteghem JL, Silue D (1992). Distribution of the mating type alleles in *Magnaporthe grisea* populations pathogenic on rice. Phytopathology.

[25] Roumen E, Levy M, Notteghem JL (1997). Characterisation of the European pathogen population of *Magnaporthe grisea* by DNA fingerprinting and pathotype analysis. Eur J Plant Pathol.

[26] Zeigler RS (1998). Recombination in *Magnaporthe grisea*. Annu Rev Phytopathol.

[27] Correll JC, Harp TL, Guerber JC, Zeigler RS, Liu B, Cartwright RD, Lee FN (2000). Characterization of *Pyricularia grisea* in the United States using independent genetic and molecular markers. Phytopathology.

[28] Noguchi MT, Yasuda N, Fujita Y (2006). Evidence of genetic exchange by parasexual recombination and genetic analysis of pathogenicity and mating type of parasexual recombinants in rice blast fungus, *Magnaporthe oryzae*. Phytopathology.

[29] Crawford MS, Chumley FG, Weaver CG, Valent B (1986). Characterization of the heterokaryotic and vegetative diploid phases of *Magnaporthe grisea*. Genetics.

[30] Genovesi AD, Magill CW (1976). Heterokaryosis and parasecuality in *Pyricularia oryzae*. Cavara. Can J Microbiol.

[31] Zeigler RS, Scott RP, Leung H, Bordeos AA, Kumar J, Nelson RJ (1997). Evidence of parasexual exchange of DNA in the rice blast fungus challenges its exclusive clonality. Phytopathology.

[32] Saleh D, Milazzo J, Adreit H, Fournier E, Tharreau D (2014). South-East Asia is the center of origin, diversity and dispersion of the rice blast fungus, *Magnaporthe oryzae*. New Phytol.

[33] Saleh D, Milazzo J, Adreit H, Tharreau D, Fournier E (2012). Asexual reproduction induces a rapid and permanent loss of sexual reproduction capacity in the rice fungal pathogen *Magnaporthe oryzae*: results of in vitro experimental evolution assays. BMC Evol Biol.

[34] Takan JP, Chipili J, Muthumeenakshi S, Talbot NJ, Manyasa EO, Bandyopadhyay R, Sere Y, Nutsugah SK, Talhinhas P, Hossain M, Brown AE, Sreenivasaprasad S (2012). *Magnaporthe oryzae* populations adapted to finger millet and rice exhibit distinctive patterns of genetic diversity, sexuality and host interaction. Mol Biotechnol.

[35] Tosa Y, Chuma I (2014). Classification and parasitic specialization of blast fungi. J Gen Plant Pathol.

[36] Couch BC, Kohn LM (2002). A multilocus gene genealogy concordant with host preference indicates segregation of a new species, *Magnaporthe oryzae*, from *M. grisea*. Mycologia.

[37] Couch BC, Fudal I, Lebrun MH, Tharreau D, Valent B, van Kim P, Notteghem JL, Kohn LM (2005). Origins of host-specific populations of the blast pathogen *Magnaporthe oryzae* in crop domestication with subsequent expansion of pandemic clones on rice and weeds of rice. Genetics.

[38] Klaubauf S, Tharreau D, Fournier E, Groenewald JZ, Crous PW, de Vries RP, Lebrun MH (2014). Resolving the polyphyletic nature of *Pyricularia* (Pyriculariaceae). Stud Mycol.

[39] Clough JM, Godfrey CRA, Hutson D, Miyamoto J (1998). The strobilurin fungicides. Fungicidal activity: chemical and biological approaches to plant protection.

[40] McKenzie KS, Andaya VC, Jodari F, Samonte SO, Oster JJ, Linquist BA, Espino LA, Mutters RG, Leinfelder-Miles MM, Wennig RL, Stogsdill JR (2016) M-209 rice: description and management guidelines. Fact Sheet Series 2016-2. University of California. Cooperative Extension. www.rice.ucanr.edu/files/234720.pdf

